# Epigenome-wide association of myocardial infarction with DNA methylation sites at loci related to cardiovascular disease

**DOI:** 10.1186/s13148-017-0353-3

**Published:** 2017-05-15

**Authors:** Masahiro Nakatochi, Sahoko Ichihara, Ken Yamamoto, Keiko Naruse, Shigeki Yokota, Hiroyuki Asano, Tatsuaki Matsubara, Mitsuhiro Yokota

**Affiliations:** 1 Statistical Analysis Section, Center for Advanced Medicine and Clinical Research, Nagoya University Hospital, Nagoya, 466-8550 Japan; 2 Department of Environmental and Preventive Medicine, Jichi Medical University School of Medicine, 3311-1 Yakushiji, Shimotsuke, 329-0498 Japan; 3 Department of Medical Biochemistry, Kurume University School of Medicine, Kurume, 830-0011 Japan; 4 Department of Internal Medicine, School of Dentistry, Aichi Gakuin University, 2-11 Suemori-dori, Chikusa-ku, Nagoya, 464-8651 Japan; 5 Department of Internal Medicine, Iwakura Hospital, Iwakura, 482-0015 Japan; 6 Department of Genome Science, School of Dentistry, Aichi Gakuin University, 2-11 Suemori-dori, Chikusa-ku, Nagoya, 464-8651 Japan

**Keywords:** DNA methylation, Myocardial infarction, Epigenome-wide association study, Cardiovascular disease, Single nucleotide polymorphism

## Abstract

**Background:**

Development of cardiovascular disease (CVD), including coronary artery disease, arrhythmia, and ischemic stroke, depends on environmental and genetic factors. To investigate the epigenetic basis of myocardial infarction (MI), we performed an epigenome-wide association study for this condition in elderly Japanese subjects. A total of 192 case subjects with MI and 192 control subjects were recruited from hospital attendees and the general population, respectively. Genome-wide DNA methylation (DNAm) profiles for DNA isolated from whole blood were obtained by analysis with an Infinium HumanMethylation450 BeadChip. The relation of DNAm sites found to be significantly associated with MI to nearby single nucleotide polymorphisms (SNPs) previously shown to be associated with CVD was assessed in the control group.

**Findings:**

Three DNAm sites (cg06642177, cg07786668, cg17218495) showed genome-wide significant associations with MI (*p* = 4.33 × 10^−8^, 3.96 × 10^−10^, and 3.77 × 10^−8^, respectively). Two of these sites (cg07786668, cg17218495) still showed such associations after adjustment for classical risk factors of MI (*p* = 1.04 × 10^−7^ and 6.60 × 10^−8^, respectively). The DNAm sites cg07786668 and cg17218495 are located in *ZFHX3* (zinc finger homeobox 3) and *SMARCA4* (SWI/SNF-related, matrix-associated, actin-dependent regulator of chromatin, subfamily a, member 4) genes, respectively. SNPs in *ZFHX3* or *SMARCA4* that were previously found to be associated with CVD were not significantly associated with these DNAm sites in our control subjects.

**Conclusions:**

We identified two DNAm sites—cg07786668 in *ZFHX3* and cg17218495 in *SMARCA4*— that are independently and significantly associated with MI. Our results suggest that the development of MI might be influenced by changes in DNAm at these sites via a pathway that differs from that affected by CVD-associated SNPs in these genes.

The Kita-Nagoya Genomic Epidemiology (KING) study, which was the source of control samples in the present study, was registered in ClinicalTrials.gov (NCT00262691) on 6 December 2005.

## Background

Cardiovascular disease (CVD) is the leading cause of mortality worldwide [1], with diabetes mellitus, hypercholesterolemia, smoking, hypertension, obesity, and physical inactivity being the primary risk factors for CVD [2]. The prevalence of CVD and its primary risk factors is increasing more rapidly in Asia than in Western countries [3]. Risk factors for CVD also include nonmodifiable characteristics such as age, male sex, ethnicity, and family history [4]. An increased understanding of the pathogenesis of CVD would be expected to help mitigate further increases in its incidence.

Genetic factors have been found to contribute to the development of CVD. Genome-wide association studies (GWASs) for CVD—including coronary artery disease (CAD) [5], arrhythmia [6, 7], and ischemic stroke [8]—have revealed many associated susceptibility genes and single nucleotide polymorphisms (SNPs). We have previously performed a candidate gene study [9], a GWAS [10], and a genome-wide linkage study [11] for myocardial infarction (MI) and thereby identified susceptibility genes for this condition. Although many SNPs associated with CVD susceptibility have been identified to date, the mechanisms by which these polymorphisms contribute to disease development have remained unclear. Furthermore, such SNPs account for only a small proportion of the heritability of CVD—that is, the portion of phenotypic variance in a population that is attributable to additive genetic factors. For example, a study of >10,000 Swedes showed that only 10.6% of the additive genetic variance of CAD was explained by 104 CAD-associated SNPs from the largest meta-analysis of this condition performed to date [5]. Improvement in the ability to predict future CVD will thus likely require the exploration of genetic biomarkers other than SNPs.

Recent progress in epigenetic epidemiology has allowed investigations of the relations among genomic coding, modifiable exposures, and manifestations of disease phenotype. DNA methylation (DNAm), a major type of epigenetic modification, is potentially an important mechanism underlying these relations [12]. DNAm plays a role in the regulation of gene expression, and DNAm status is affected by the environment [13], with variation in such status having been associated with age [14] and smoking [15]. Gender and ethnicity also contribute to DNAm status [16]. Given that differential DNAm might explain differences in disease susceptibility or phenotype, DNAm has the potential to serve as a novel genetic biomarker of exposure or of disease risk or progression [17, 18].

Epigenome-wide association studies (EWASs) that explore DNAm sites associated with phenotypes have recently revealed that DNAm status at some such sites in blood samples is associated with risk factors for CVD such as body mass index (BMI) [19], blood lipid levels [20], plasma resistin concentration [21], and type 2 diabetes [22]. Some studies have also found DNAm status in blood samples to be associated with CVD itself [23]. Given that DNAm and CVD are both affected by many factors such as age, sex, and ethnicity, however, it is difficult to identify DNAm sites that are associated with CVD independently of such factors. Further studies in which case and control subjects are matched in age and comprise a single sex and ethnicity are needed in order to elucidate the relation of DNAm to CVD.

We have now measured genome-wide DNAm status for DNA samples prepared from whole blood of patients with MI attending hospitals in Japan [9–11] and of the elderly Japanese participants of the Kita-Nagoya Genomic Epidemiology (KING) study [24–26] and thereby performed an EWAS for MI in Japanese men. With our control subjects, we then assessed the relation of DNAm sites identified in our study to nearby SNPs previously found to be associated with CVD in GWASs.

## Methods

### Study subjects

We performed a cross-sectional case-control study to examine the association of DNAm status at various sites with MI. A total of 192 male cases and 192 male controls were enrolled. All subjects were ≥55 years old, and the two groups were matched in age (within 5 years). The 192 men with MI were randomly selected from individuals previously recruited through participating hospitals in Japan [9–11]. The diagnosis of MI was based on typical electrocardiographic changes and increased serum activities of enzymes including creatine kinase, aspartate aminotransferase, and lactate dehydrogenase; it was confirmed by the presence of a wall motion abnormality on left ventriculography and attendant stenosis in any of the major coronary arteries. The 192 controls were nondiabetic men randomly selected from participants of the ongoing KING study (ClinicalTrials.gov identifier, NCT00262691) [24–26] and whose data were used in a previous study [21]. The control subjects had no history of CAD or other CVD. Subjects with a systolic blood pressure of ≥140 mmHg or a diastolic blood pressure of ≥90 mmHg, or those who were currently taking antihypertensive medication were categorized as having hypertension. Hyperlipidemia was defined as a serum concentration of total cholesterol of ≥5.68 mmol/L or the taking of lipid-lowering drugs. Diabetes was defined as a fasting plasma glucose concentration of ≥7.0 mmol/L, a hemoglobin A_1c_ level (measured according to the Japan Diabetes Society method) of ≥6.5%, or current treatment for diabetes. The characteristics of the subjects are shown in Table 1. Venous blood was collected from subjects in the fasted condition into tubes containing EDTA, and genomic DNA was isolated with the use of a kit (Qiagen, Chatsworth, CA).

**Table 1:** Characteristics of the study subjects

Characteristic	Controls	Cases	*p*
	(*n* = 192)	(*n* = 192)	
Male, *n* (%)	192 (100%)	192 (100%)	1.000
Age (years)	65.8 ± 6.0	65.9 ± 6.4	0.915
BMI (kg/m^2^)	23.1 ± 2.5	23.9 ± 2.7	0.001*
Current smoker, *n* (%)	43 (22.4%)	83 (43.2%)	2.01 × 10^−5^*
Hypertension, *n* (%)	106 (55.2%)	113 (58.9%)	0.536
Diabetes mellitus, *n* (%)	0 (0%)	77 (40.1%)	8.82 × 10^−28^*
Hyperlipidemia, *n* (%)	75 (39.1%)	106 (55.2%)	0.002*
ST segment elevation myocardial infarction, *n* (%)		192 (100%)	

Continuous data are means ± SD. Differences in characteristics between case and control groups were evaluated by Student’s *t* test or Fisher’s exact test

*Significance (*p* < 0.05)

### DNAm analysis

Genomic DNA was processed with the use of an EZ-96 DNA Methylation Kit (Zymo Research, Orange, CA), which combines bisulfite conversion and DNA cleanup in a 96-well plate. Genome-wide DNAm profiles were obtained for case and control subjects at the same time by analysis with an Infinium HumanMethylation450 BeadChip (Illumina, San Diego, CA). For the EWAS analysis, we applied a recently developed correction method to reduce technical bias in the DNAm array data [27, 28]. Marker intensities were normalized by quantile normalization. DNAm level was quantified as a β value. One sample from the control group was excluded from further analysis because the DNAm profile was not detected as a result of mixing with air bubbles during sample loading, whereas a sample from the case group was excluded because of mismatched sex based on the DNAm profile. We performed principal component analysis to quantify latent structure in the data, including batch effects. We estimated the cell type composition for each sample with the estimateCellCounts function [29] in minfi of the R package. These estimated parameters were used in the association analysis as covariates. For each sample, probes with a detection *p* value of ≥1 × 10^−16^ were assigned not-detected status, and DNAm level with not-detected status was set to a missing value. Each sample had <10% of all probes with not-detected status. We removed nonautosomal probes as well as probes with a not-detected status in ≥2% of the samples. We further excluded probes previously found to be cross-reactive (≥47 bases) [30]. Probes containing SNPs have been found to influence the assessment of DNAm status with the Infinium HumanMethylation450 array [31], and an effect of CpG SNPs on DNAm has also been reported [32]. We therefore filtered out probes that contain SNPs with a minor allele frequency (MAF) of >0.01 based on 1000 Genomes ASN [30] in order to reduce the frequency of false positives. Finally, 191 case subjects and 191 control subjects as well as 348,595 DNAm sites remained for the EWAS analysis.

### Genotyping of *ZFHX3* and *SMARCA4* SNPs

All blood-derived DNA samples evaluated for DNAm status in the control group were also genotyped with the use of an Illumina HumanOmniExpress-12 BeadChip [21]. The data were subjected to quality control procedures, by which SNPs with a call rate of <0.98, a MAF of <0.01, or a Hardy-Weinberg equilibrium *p* value of <1 × 10^−6^ were filtered out. Of the *ZFHX3* (zinc finger homeobox 3) SNPs that passed quality control, three polymorphisms (rs7193343, rs2106261, rs879324) were previously found to be associated with CVD [6–8] and so were subjected to further analysis. None of the *SMARCA4* (SWI/SNF-related, matrix-associated, actin-dependent regulator of chromatin, subfamily a, member 4) SNPs that passed quality control had previously been found to be associated with CVD, but rs3786725 was shown to be in strong linkage disequilibrium with rs1122608 (*r* ^2^ = 0.956, calculated from Asian samples of the 1000 Genomes Project phase I), which was reported to be associated with CVD [33], and so was subjected to further analysis.

### Statistical analysis

History of diseases and other clinical variables were compared between the case and control groups with Student’s *t* test or Fisher’s exact test. The association of DNAm status at each DNAm site with MI was assessed with a general linear model (GLM); the dependent variable was DNAm status at each site, and the independent variables included MI label (case = 1, control = 0) and covariates. We applied two types of model to assess the association of DNAm at each site with MI. The covariates in model 1 comprised age, the first 30 principal component scores calculated from Infinium 450K assay control probes, the first five principal component scores calculated from the residuals after adjustment for technical and biological factors, and the cell type composition of samples. The covariates in model 2 included those of model 1 as well as BMI, smoking status (noncurrent smoker = 0, current smoker = 1), and history (0 = no history, 1 = positive history) of diabetes, hypertension, and hyperlipidemia. We corrected the association results for the genomic control inflation factor. The relation between DNAm status at two DNAm sites was assessed with Pearson’s correlation coefficient.

To test the association of DNAm status at each DNAm site with each SNP in the control group, we adopted a GLM with adjustment for covariates used in model 1; the dependent variable was DNAm status at each site, and independent variables included the genotype of each SNP and the covariates used in model 1. We coded genotypes as 0, 1, or 2 on the basis of the number of minor alleles.

For the EWAS analysis, the significance level α was determined by dividing 0.05 by the number of DNAm sites for Bonferroni correction (α = 0.05/348,595 = 1.43 × 10^−7^). A *p* value of <0.05 was considered nominally significant. All statistical analysis was performed with the R project (version 3.3.0, http://www.r-project.org/).

## Results

### Characteristics of the study subjects

The baseline characteristics of the study subjects are shown in Table 1. Mean ± SD values of age in the case and control groups were 65.9 ± 6.4 and 65.8 ± 6.0 years, respectively. BMI as well as the frequency of current smokers, diabetes mellitus, and hyperlipidemia were significantly higher in the case group than in the control group.

### Association analysis for DNAm status and MI

**Fig. 1:**
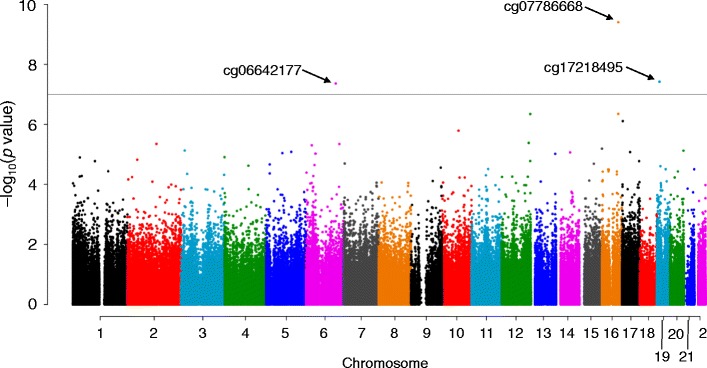
Manhattan plot for EWAS analysis of DNAm and MI. The *horizontal line* represents the genome-wide significance level (*α* = 1.43 × 10^−7^). The *p* values were corrected for genomic control

**Fig. 2:**
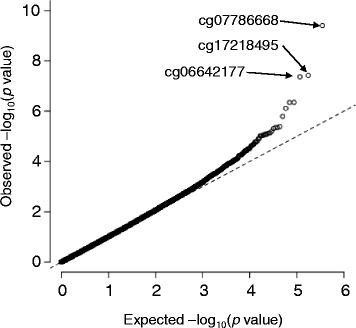
Quantile-quantile (Q-Q) plot of observed versus expected –log_10_(*p* value) for tests of association between DNAm sites and MI. The *p* values were corrected for genomic control

**Fig. 3:**
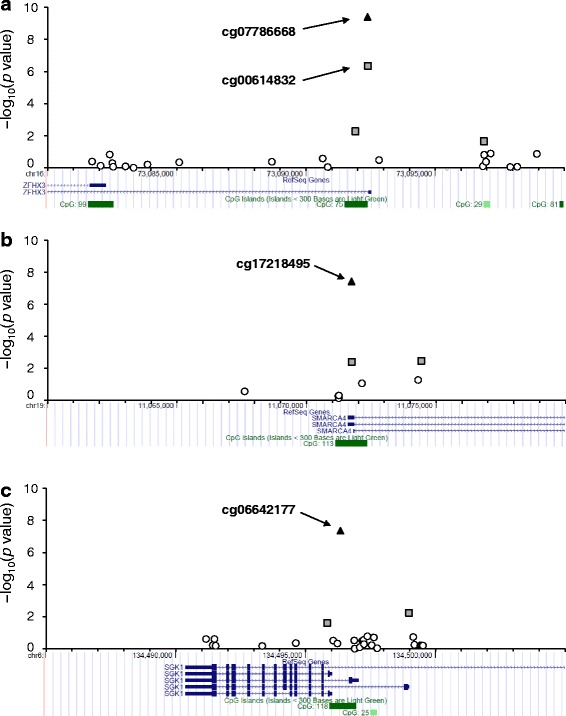
Regional plots for the three DNAm sites that showed a genome-wide significant association with MI. The vertical axis represents –log_10_(*p* value) for assessment of the association of cg07786668 (**a**), cg17218495 (**b**), or cg06642177 (**c**) with MI. The DNAm sites that achieved a genome-wide significance level (*α* = 1.43 × 10^−7^) are shown as *black triangles*, those that achieved a nominal significance level (*α* = 0.05) are shown as *gray squares*, and other DNAm sites are shown as *open circles*

We performed genome-wide DNAm profiling for whole-blood DNA from 192 case and 192 control subjects. After initial processing, 191 case and 191 control subjects as well as 348,595 DNAm sites remained for subsequent analysis. We initially performed an association analysis for DNAm status at each site and MI with model 1. Weak inflation in low *p* values was observed (*λ* = 1.04). We therefore corrected the association results for the genomic control inflation factor. Three DNAm sites (cg07786668, cg17218495, cg06642177) achieved a genome-wide significance level (Fig. 1 and Table 2). These sites were also detected as outliers in a quantile-quantile (Q-Q) plot of −log_10_(*p*) for the 348,595 tests of association between DNAm status and MI (Fig. 2). Regional plots of the flanking regions of the three DNAm sites are shown in Fig. 3. The sites are located within CpG islands of *ZFHX3*, *SMARCA4*, and *SGK1* (serum/glucocorticoid-regulated kinase 1), respectively. The regional plot of cg07786668 contains another DNAm site, cg00614832, that showed a nominally significant association with MI (*p* = 4.48 × 10^−7^). The methylation status of these two DNAm sites showed a significant positive correlation (*r* = 0.395, *p* = 1.01 × 10^−15^).

**Table 2:** Association analysis for MI and genome-wide significant DNAm sites

DNAm site	Chr	Position (bp)	Nearest gene	Relation to CpG island	Model 1		Model 2	
					Effect ± SE	*p*	Effect ± SE	*p*
cg06642177	6	134,496,341	*SGK1*	CpG island	0.023 ± 0.004	4.33 × 10^−8^*	0.022 ± 0.004	7.47 × 10^−7^
cg07786668	16	73,092,391	*ZFHX3*	CpG island	0.018 ± 0.003	3.96 × 10^−10^*	0.016 ± 0.003	1.04 × 10^−7^*
cg17218495	19	11,071,743	*SMARCA4*	CpG island	0.015 ± 0.003	3.77 × 10^−8^*	0.015 ± 0.003	6.60 × 10^−8^*

The Effect and *p* values were calculated with a GLM, with the Effect values representing change in β value per change from control to case. The *p* values were corrected for genomic control

*Chr* chromosome

*Genome-wide significance (*p* < 1.43 × 10^−7^)

We also evaluated the relation of the three DNAm sites cg07786668, cg17218495, and cg06642177 to MI with model 2, which adjusts for covariates including other risk factors. Two of the three sites, cg07786668 and cg17218495, again showed a genome-wide significant association with MI (Table 2).

We explored potential functional annotations in the ENCODE database for the genomic regions surrounding the DNAm sites cg07786668, cg17218495, and cg06642177 with the use of RegulomeDB [34]. The RegulomeDB score for each of these sites was 2b with transcription factor binding site, any motif; DNase footprint; and DNase peak.

### Association analysis of DNAm status and SNPs

We next performed an association analysis with control subjects for DNAm status at the *ZFHX3* DNAm sites cg07786668 and cg00614832 and the *SMARCA4* DNAm site cg17218495 and for SNPs in *ZFHX3* and *SMARCA4* that were previously found to be associated with CVD. No significant association between these SNPs and the corresponding DNAm sites was detected (Table 3).

**Table 3:** Association analysis for SNPs and DNAm sites located in *ZFHX3* and *SMARCA4* for the control group

SNP	Chromosome	Position (bp)	Strand	Allele (minor/major)	MAF (%)	DNAm site	Effect ± SE	*p*
*ZFHX3*								
rs7193343	16	73,029,160	+	C/T	40.4	cg07786668	−0.0011 ± 0.0011	0.307
						cg00614832	0.0004 ± 0.0013	0.755
rs2106261	16	73,051,620	+	T/C	33.1	cg07786668	−0.0016 ± 0.0011	0.176
						cg00614832	0.0004 ± 0.0014	0.774
rs879324	16	73,068,678	+	A/G	38.3	cg07786668	−0.0015 ± 0.0011	0.186
						cg00614832	0.0005 ± 0.0014	0.718
*SMARCA4*								
rs3786725^a^	19	11,166,827	+	A/G	11.2	cg17218495	0.0008 ± 0.0011	0.461

The Effect and *p* values were calculated with a GLM. The Effect value represents the change in β-value per minor allele copy for the SNP

^a^This SNP is in linkage disequilibrium with rs1122608 in *SMARCA4* (*r* ^2^ = 0.956 based on Asian samples of the 1000 Genomes Project phase I)

## Discussion

We have here performed an EWAS for MI in aged Japanese men with the use of the Infinium HumanMethylation450 array. Our analysis of 191 case and 191 control subjects detected genome-wide significant associations of MI with three DNAm sites (cg07786668, cg17218495, cg06642177). Several EWASs have recently revealed that age, sex, and ethnicity are strongly associated with the methylation status of many DNAm sites in blood samples [16, 35–37]. Such background associations can hinder the performance of EWASs for other phenotypes. Our study subjects were recruited from only Japanese men, and the cases and controls were matched in age (within 5 years). The results of our study are therefore expected to be more reliable than those of studies with less homogeneous subject populations. Recent EWASs have also revealed that DNAm status at some DNAm sites in blood samples is associated with classical risk factors for CVD such as BMI [19], blood lipid levels [20], and type 2 diabetes [22]. In our study, two DNAm sites, cg07786668 and cg17218495, remained significantly associated with MI at the genome-wide level after adjustment for these classical risk factors of CVD, suggesting that these two sites contribute independently to the presence of MI. The three DNAm sites identified in our study are located in CpG islands within the noncoding regions of *ZFHX3*, *SMARCA4*, and *SGK1*. Furthermore, RegulomeDB scores for each of these DNAm sites were 2b with transcription factor binding site, any motif; DNase footprint; and DNase peak. These scores indicate that the DNAm sites are located in regulatory regions of the corresponding genes.

The DNAm site cg07786668 showed the most significant association with MI in model 1. The DNAm status of sites cg07786668 and cg00614832 was positively correlated, possibly reflecting a common biological process in case subjects with MI. These DNAm sites are located in *ZFHX3*, which encodes the transcription factor ZFHX3 and is widely expressed, with its expression having been detected in all 16 tissues covered by the Body Map 2.0 project [38]. *ZFHX3* has also been shown to be associated with susceptibility to several CVD-related phenotypes. GWASs have thus identified associations between SNPs in this gene and atrial fibrillation [6, 7] and cardioembolic stroke [7, 8]. Our results now provide further evidence that *ZFHX3* is a susceptibility gene for CVD. Recent findings suggest that a disease might be influenced by disease-associated SNPs via changes in DNAm near the SNPs [21, 39]. However, no significant association was apparent in our control subjects between DNAm status at cg07786668 or cg00614832 and *ZFHX3* SNPs (rs7193343, rs2106261, rs879324) that had previously been associated with CVD. These DNAm sites may therefore contribute to the development of MI via a biological pathway different from that affected by the SNPs.

We also found that DNAm site cg17218495 located in *SMARCA4* showed a genome-wide significant association with MI. *SMARCA4* encodes the catalytic subunit of the SWI/SNF chromatin-remodeling complex and influences transcriptional regulation by disrupting histone-DNA contacts in an ATP-dependent manner [40]. Overexpression or knockdown of SMARCA4 was recently shown to affect inhibition of vascular smooth muscle cell proliferation by hydrogen sulfide [41]. *SMARCA4* has also been shown to be associated with susceptibility to CVD. Previous GWASs thus identified associations of the SNP rs1122608 in this gene with early-onset MI [33] and CAD [42, 43]. However, we did not detect a significant association of DNAm status at cg17218495 with the *SMARCA4* SNP rs3786725, which is in strong linkage disequilibrium with rs1122608, in our control subjects. This DNAm site may thus also contribute to the development of MI via a pathway different from that affected by rs1122608.

The DNAm site cg06642177 located in *SGK1* showed a genome-wide significant association with MI in model 1. Candidate gene studies revealed that the *SGK1* SNPs rs1057293 and rs1743966 were both associated with hypertension [44] and ischemic stroke [45]. In our study, however, this DNAm site did not show a genome-wide significant association with MI in model 2, which adjusts for classical risk factors of MI.

DNAm status in blood samples has also been found to be associated with CVD itself [23], and two EWASs for MI were recently performed [46, 47]. Rask-Andersen et al. thus performed an EWAS for a history of MI in a population cohort from northern Sweden [47]. They found that 211 DNAm sites in 196 genes were associated with a history of MI, with 42 of these genes having previously been shown to be related to CVD, cardiac function, cardiogenesis, or recovery from ischemic injury. The DNAm sites cg07786668 and cg17218495 identified in our study were not included among the 211 DNAm sites of this previous EWAS. However, the results of the two studies are consistent in that DNAm sites located around genes associated with susceptibility to CVD were found to be associated with MI. In particular, Rask-Andersen et al. found that the DNAm site cg05896042 located near *SMARCA4* was associated with a history of MI and was not significantly associated with rs1122608 located near this gene. In our study, the DNAm site cg05896042 was not significantly associated with MI (*p* = 0.231 in model 1). The two studies thus indicate that DNAm sites located around *SMARCA4* are related to MI, but they differ with regard to the specific DNAm sites, possibly reflecting differences in other factors such as ethnicity. Guarrera et al. performed an EWAS for MI in an Italian population in a case-control setting [46]. They focused on differentially methylated regions that comprise clusters of DNAm sites located around genes, and they found that such regions within *ZBTB12* and long interspersed nuclear element–1 were associated with MI. Global alterations at DNAm sites following MI have previously been demonstrated [48]. Rask-Andersen et al. also detected some degree of inflation in low *p* values (*λ* = 1.44) [47], and we observed weak inflation in *p* values (*λ* = 1.04).

There are several limitations to the present study: (i) The study was exploratory in nature and our findings were not validated in replication cohorts. Future studies will therefore be necessary to validate our findings in independent cohorts. (ii) The EWAS was performed with the Infinium HumanMethylation450 array, with the consequence that not all DNAm sites in the human genome were inspected. Further insight into the association of DNAm sites with MI will require fine-mapping analysis with bisulfite sequencing. (iii) The study is cross-sectional in nature and therefore does not establish a cause-and-effect relation between DNAm level at DNAm sites and MI. Future studies are thus necessary to evaluate such relations in prospective cohorts. (iv) We studied only male subjects, with the result that our findings will require confirmation in female subjects. (v) We measured DNAm in whole-blood cells as a surrogate for heart tissue. Given that DNAm status at specific sites may be tissue dependent, our findings may not reflect MI-associated changes in DNAm in heart tissue.

## Conclusions

We have revealed genome-wide significant associations of MI with DNAm status at three DNAm sites—cg07786668 in *ZFHX3*, cg17218495 in *SMARCA4*, and cg06642177 in *SGK1*—in blood samples, with *ZFHX3* and *SMARCA4* having previously been identified as susceptibility genes for CVD. Although SNPs located in these genes have been found to be associated with CVD, DNAm status at the DNAm sites identified here was not associated with these SNPs. Our results thus suggest the possibility that these DNAm sites are independently related to the development of MI.

## Abbreviations

BMI: Body mass index
CAD: Coronary artery disease
CVD: Cardiovascular disease
DNAm: DNA methylation
EWAS: Epigenome-wide association study
GLM: General linear model
GWAS: Genome-wide association study
KING: Kita-Nagoya Genomic Epidemiology
MAF: Minor allele frequency
MI: Myocardial infarction
SNP: Single nucleotide polymorphism

## References

[1] Murray CJ, Lopez AD (1997). Alternative projections of mortality and disability by cause 1990-2020: Global Burden of Disease Study. Lancet.

[2] Greenland P, Alpert JS, Beller GA, Benjamin EJ, Budoff MJ, Fayad ZA, Foster E, Hlatky MA, Hodgson JM, Kushner FG (2010). ACCF/AHA guideline for assessment of cardiovascular risk in asymptomatic adults: a report of the American College of Cardiology Foundation/American Heart Association Task Force on Practice Guidelines. Circulation.

[3] Kitakaze M (2015). Trends in characteristics of CVD in Asia and Japan: the importance of epidemiological studies and beyond. J Am Coll Cardiol.

[4] D'Agostino RB, Pencina MJ, Massaro JM, Coady S (2013). Cardiovascular disease risk assessment: insights from Framingham. Glob Heart.

[5] Consortium CAD, Deloukas P, Kanoni S, Willenborg C, Farrall M, Assimes TL, Thompson JR, Ingelsson E, Saleheen D, Erdmann J (2013). Large-scale association analysis identifies new risk loci for coronary artery disease. Nat Genet.

[6] Benjamin EJ, Rice KM, Arking DE, Pfeufer A, van Noord C, Smith AV, Schnabel RB, Bis JC, Boerwinkle E, Sinner MF (2009). Variants in ZFHX3 are associated with atrial fibrillation in individuals of European ancestry. Nat Genet.

[7] Gudbjartsson DF, Holm H, Gretarsdottir S, Thorleifsson G, Walters GB, Thorgeirsson G, Gulcher J, Mathiesen EB, Njolstad I, Nyrnes A (2009). A sequence variant in ZFHX3 on 16q22 associates with atrial fibrillation and ischemic stroke. Nat Genet.

[8] Traylor M, Farrall M, Holliday EG, Sudlow C, Hopewell JC, Cheng YC, Fornage M, Ikram MA, Malik R, Bevan S (2012). Genetic risk factors for ischaemic stroke and its subtypes (the METASTROKE collaboration): a meta-analysis of genome-wide association studies. Lancet Neurol.

[9] Yamada Y, Izawa H, Ichihara S, Takatsu F, Ishihara H, Hirayama H, Sone T, Tanaka M, Yokota M (2002). Prediction of the risk of myocardial infarction from polymorphisms in candidate genes. N Engl J Med.

[10] Takeuchi F, Yokota M, Yamamoto K, Nakashima E, Katsuya T, Asano H, Isono M, Nabika T, Sugiyama T, Fujioka A (2012). Genome-wide association study of coronary artery disease in the Japanese. Eur J Hum Genet.

[11] Ichihara S, Yamamoto K, Asano H, Nakatochi M, Sukegawa M, Ichihara G, Izawa H, Hirashiki A, Takatsu F, Umeda H (2013). Identification of a glutamic acid repeat polymorphism of ALMS1 as a novel genetic risk marker for early-onset myocardial infarction by genome-wide linkage analysis. Circ Cardiovasc Genet.

[12] Zhong J, Agha G, Baccarelli AA (2016). The role of DNA methylation in cardiovascular risk and disease: methodological aspects, study design, and data analysis for epidemiological studies. Circ Res.

[13] Kaminsky ZA, Tang T, Wang SC, Ptak C, Oh GH, Wong AH, Feldcamp LA, Virtanen C, Halfvarson J, Tysk C (2009). DNA methylation profiles in monozygotic and dizygotic twins. Nat Genet.

[14] Rakyan VK, Down TA, Maslau S, Andrew T, Yang TP, Beyan H, Whittaker P, McCann OT, Finer S, Valdes AM (2010). Human aging-associated DNA hypermethylation occurs preferentially at bivalent chromatin domains. Genome Res.

[15] Breitling LP, Yang R, Korn B, Burwinkel B, Brenner H (2011). Tobacco-smoking-related differential DNA methylation: 27 K discovery and replication. Am J Hum Genet.

[16] Zhang FF, Cardarelli R, Carroll J, Fulda KG, Kaur M, Gonzalez K, Vishwanatha JK, Santella RM, Morabia A (2011). Significant differences in global genomic DNA methylation by gender and race/ethnicity in peripheral blood. Epigenetics.

[17] Mill J, Heijmans BT (2013). From promises to practical strategies in epigenetic epidemiology. Nat Rev Genet.

[18] Rakyan VK, Down TA, Balding DJ, Beck S (2011). Epigenome-wide association studies for common human diseases. Nat Rev Genet.

[19] Dick KJ, Nelson CP, Tsaprouni L, Sandling JK, Aissi D, Wahl S, Meduri E, Morange PE, Gagnon F, Grallert H (2014). DNA methylation and body-mass index: a genome-wide analysis. Lancet.

[20] Pfeiffer L, Wahl S, Pilling LC, Reischl E, Sandling JK, Kunze S, Holdt LM, Kretschmer A, Schramm K, Adamski J (2015). DNA methylation of lipid-related genes affects blood lipid levels. Circ Cardiovasc Genet.

[21] Nakatochi M, Ichihara S, Yamamoto K, Ohnaka K, Kato Y, Yokota S, Hirashiki A, Naruse K, Asano H, Izawa H (2015). Epigenome-wide association study suggests that SNPs in the promoter region of RETN influence plasma resistin level via effects on DNA methylation at neighbouring sites. Diabetologia.

[22] Chambers JC, Loh M, Lehne B, Drong A, Kriebel J, Motta V, Wahl S, Elliott HR, Rota F, Scott WR (2015). Epigenome-wide association of DNA methylation markers in peripheral blood from Indian Asians and Europeans with incident type 2 diabetes: a nested case-control study. Lancet Diabetes Endocrinol.

[23] Muka T, Koromani F, Portilla E, O'Connor A, Bramer WM, Troup J, Chowdhury R, Dehghan A, Franco OH (2016). The role of epigenetic modifications in cardiovascular disease: a systematic review. Int J Cardiol.

[24] Nakatochi M, Miyata S, Tanimura D, Izawa H, Asano H, Murase Y, Kato R, Ichihara S, Naruse K, Matsubara T (2011). The ratio of adiponectin to homeostasis model assessment of insulin resistance is a powerful index of each component of metabolic syndrome in an aged Japanese population: results from the KING Study. Diabetes Res Clin Pract.

[25] Asano H, Izawa H, Nagata K, Nakatochi M, Kobayashi M, Hirashiki A, Shintani S, Nishizawa T, Tanimura D, Naruse K (2010). Plasma resistin concentration determined by common variants in the resistin gene and associated with metabolic traits in an aged Japanese population. Diabetologia.

[26] Tanimura D, Shibata R, Izawa H, Hirashiki A, Asano H, Murase Y, Miyata S, Nakatochi M, Ouchi N, Ichihara S (2011). Relation of a common variant of the adiponectin gene to serum adiponectin concentration and metabolic traits in an aged Japanese population. Eur J Hum Genet.

[27] Lehne B, Drong AW, Loh M, Zhang W, Scott WR, Tan ST, Afzal U, Scott J, Jarvelin MR, Elliott P (2015). A coherent approach for analysis of the Illumina HumanMethylation450 BeadChip improves data quality and performance in epigenome-wide association studies. Genome Biol.

[28] Shiwa Y, Hachiya T, Furukawa R, Ohmomo H, Ono K, Kudo H, Hata J, Hozawa A, Iwasaki M, Matsuda K (2016). Adjustment of cell-type composition minimizes systematic bias in blood DNA methylation profiles derived by dna collection protocols. PLoS One.

[29] Jaffe AE, Irizarry RA (2014). Accounting for cellular heterogeneity is critical in epigenome-wide association studies. Genome Biol.

[30] Chen YA, Lemire M, Choufani S, Butcher DT, Grafodatskaya D, Zanke BW, Gallinger S, Hudson TJ, Weksberg R (2013). Discovery of cross-reactive probes and polymorphic CpGs in the Illumina Infinium HumanMethylation450 microarray. Epigenetics.

[31] Price ME, Cotton AM, Lam LL, Farre P, Emberly E, Brown CJ, Robinson WP, Kobor MS (2013). Additional annotation enhances potential for biologically-relevant analysis of the Illumina Infinium HumanMethylation450 BeadChip array. Epigenetics Chromatin.

[32] Dayeh TA, Olsson AH, Volkov P, Almgren P, Ronn T, Ling C (2013). Identification of CpG-SNPs associated with type 2 diabetes and differential DNA methylation in human pancreatic islets. Diabetologia.

[33] Kathiresan S, Voight BF, Purcell S, Musunuru K, Ardissino D, Mannucci PM, Anand S, Engert JC, Samani NJ, Schunkert H (2009). Genome-wide association of early-onset myocardial infarction with single nucleotide polymorphisms and copy number variants. Nat Genet.

[34] Boyle AP, Hong EL, Hariharan M, Cheng Y, Schaub MA, Kasowski M, Karczewski KJ, Park J, Hitz BC, Weng S (2012). Annotation of functional variation in personal genomes using RegulomeDB. Genome Res.

[35] Langevin SM, Houseman EA, Christensen BC, Wiencke JK, Nelson HH, Karagas MR, Marsit CJ, Kelsey KT (2011). The influence of aging, environmental exposures and local sequence features on the variation of DNA methylation in blood. Epigenetics.

[36] Bell JT, Tsai PC, Yang TP, Pidsley R, Nisbet J, Glass D, Mangino M, Zhai G, Zhang F, Valdes A (2012). Epigenome-wide scans identify differentially methylated regions for age and age-related phenotypes in a healthy ageing population. PLoS Genet.

[37] Liu J, Morgan M, Hutchison K, Calhoun VD (2010). A study of the influence of sex on genome wide methylation. PLoS One.

[38] Flicek P, Ahmed I, Amode MR, Barrell D, Beal K, Brent S, Carvalho-Silva D, Clapham P, Coates G, Fairley S (2013). Ensembl 2013. Nucleic Acids Res.

[39] Kato N, Loh M, Takeuchi F, Verweij N, Wang X, Zhang W, Kelly TN, Saleheen D, Lehne B, Mateo Leach I (2015). Trans-ancestry genome-wide association study identifies 12 genetic loci influencing blood pressure and implicates a role for DNA methylation. Nat Genet.

[40] Medina PP, Carretero J, Ballestar E, Angulo B, Lopez-Rios F, Esteller M, Sanchez-Cespedes M (2005). Transcriptional targets of the chromatin-remodelling factor SMARCA4/BRG1 in lung cancer cells. Hum Mol Genet.

[41] Li L, Liu D, Bu D, Chen S, Wu J, Tang C, Du J, Jin H (1833). Brg1-dependent epigenetic control of vascular smooth muscle cell proliferation by hydrogen sulfide. Biochim Biophys Acta.

[42] Schunkert H, Konig IR, Kathiresan S, Reilly MP, Assimes TL, Holm H, Preuss M, Stewart AF, Barbalic M, Gieger C (2011). Large-scale association analysis identifies 13 new susceptibility loci for coronary artery disease. Nat Genet.

[43] Dichgans M, Malik R, Konig IR, Rosand J, Clarke R, Gretarsdottir S, Thorleifsson G, Mitchell BD, Assimes TL, Levi C (2014). Shared genetic susceptibility to ischemic stroke and coronary artery disease: a genome-wide analysis of common variants. Stroke.

[44] Busjahn A, Aydin A, Uhlmann R, Krasko C, Bahring S, Szelestei T, Feng Y, Dahm S, Sharma AM, Luft FC, Lang F (2002). Serum- and glucocorticoid-regulated kinase (SGK1) gene and blood pressure. Hypertension.

[45] Dahlberg J, Smith G, Norrving B, Nilsson P, Hedblad B, Engstrom G, Lovkvist H, Carlson J, Lindgren A, Melander O (2011). Genetic variants in serum and glucocortocoid regulated kinase 1, a regulator of the epithelial sodium channel, are associated with ischaemic stroke. J Hypertens.

[46] Guarrera S, Fiorito G, Onland-Moret NC, Russo A, Agnoli C, Allione A, Di Gaetano C, Mattiello A, Ricceri F, Chiodini P (2015). Gene-specific DNA methylation profiles and LINE-1 hypomethylation are associated with myocardial infarction risk. Clin Epigenetics.

[47] Rask-Andersen M, Martinsson D, Ahsan M, Enroth S, Ek WE, Gyllensten U, Johansson A (2016). Epigenome-wide association study reveals differential DNA methylation in individuals with a history of myocardial infarction. Hum Mol Genet.

[48] Kim M, Long TI, Arakawa K, Wang R, Yu MC, Laird PW (2010). DNA methylation as a biomarker for cardiovascular disease risk. PLoS One.

